# Pediatric fluoroquinolone prescription in South Korea before and after a regulatory intervention: A nationwide study, 2007-2015

**DOI:** 10.1371/journal.pone.0176420

**Published:** 2017-05-17

**Authors:** Seung Yeon Song, Joo Hee Shin, Su Yeong Hyeon, Donguk Kim, Won Ku Kang, Soo-Han Choi, Yae-Jean Kim, Eun Young Kim

**Affiliations:** 1 Department of Health, Social and Clinical Pharmacy, Chung-Ang University College of Pharmacy, Seoul, South Korea; 2 Department of Clinical Pharmacy, Chungnam National University College of Pharmacy, Daejun, South Korea; 3 Department of Statistics, Sungkyunkwan University, Seoul, South Korea; 4 Department of Pediatrics, Hallym University Dongtan Sacred Heart Hospital, Gyeonggi-do, South Korea; 5 Department of Pediatrics, Samsung Medical Centre, Sungkyunkwan University School of Medicine, Seoul, South Korea; University of North Carolina at Chapel Hill School of Dentistry, UNITED STATES

## Abstract

**Objective:**

To investigate the impact of national implementation of age restriction on fluoroquinolone prescription in children and adolescents.

**Methods:**

Data collected from the database of Health Insurance Review and Assessment Service in South Korea, a national health insurance system to analyze fluoroquinolone prescribing practice in children and adolescents younger than 18 years, between 2007 and 2015. The age restriction was implemented in December 2009. The annual prescription rate of FQ per 100,000 person-years was calculated and an autoregressive model was used to predict the prescription pattern if an intervention had not occurred.

**Results:**

A total of 505,859 children received systemic fluoroquinolone during the study period—297,054 ciprofloxacin, and 208,805 levofloxacin. After implementation of the drug utilization review program, the annual prescription rate for ciprofloxacin declined by 97.5% (from 840 to 21 per 100,000 person-years, P < 0.001), and for levofloxacin by 96.4% (from 598 to 11 per 100,000 person-years, P < 0.001). The decline was more dramatic in the outpatient setting than in the inpatient setting for both drugs.

**Conclusion:**

The dramatic and sustained decline in prescription number and change in prescription pattern after the regulatory action suggests that the implementation under drug utilization review program was successful in controlling excessive and inappropriate use of fluoroquinolones in children, possibly guiding towards more judicious and selective prescription behavior.

## Introduction

Pediatric use of FQs has been restricted due to early findings of FQ-associated weight-bearing joint damage in juvenile animals [1–4]. However, the off-label pediatric use of FQ outside the regulatory boundaries is thought to be common and increasing worldwide [5–9]. Development of resistance to other antibiotics is thought to be the main driving factor behind the increasing use of FQs in children [10–15]. However, FQ itself is also associated with the rapid development of resistance, and thus, these agents should be used with caution and only when necessary [2, 16, 17]. A worldwide emergence of quinolone resistance has been observed, especially with *Escherichia coli* isolates, and this phenomenon is thought to be most prominent in the Asia–Pacific region [18–24]. Therefore, inappropriate and excessive use of FQs in children, especially in the ambulatory setting, is of concern in this era of increased antimicrobial resistance. Interventions have been often implemented on the individual hospital level to control the inappropriate use of antibiotics and restrict the use of certain broad-spectrum antibiotics. In South Korea, an intervention was implemented on a national level to control fluoroquinolone use of in children.

South Korea has a unique Drug Utilization Review (DUR) system that is concurrent and provides real-time information to prescribers and pharmacists [25]. It uses a predefined DUR criteria that include a list of medications that are contraindicated in pregnant women, drugs with drug–drug interactions, and drugs with age restrictions. After its initial establishment in 2004, 58 drugs were added to the list of contraindicated drugs in pediatrics in December 2009, which included the fluoroquinolone (FQ) class of antibiotics [26]. When a contraindicated drug is prescribed, an alert appears; at this point, the prescriber may change the prescription or provide a reason to justify the use and continue with the prescription. When a contraindicated drug is dispensed, a similar warning appears, and the pharmacist should contact the prescriber and confirm the prescription.

Before December 2009, there were no regulatory restrictions on FQ use in children and the Korean regulatory agencies reimbursed the use of FQs for patients of all ages. Such measure was in contrast to other countries where the regulatory approval status of FQs in children was expanded to allow the use in a wider range of indications, which was supported by the increasing availability of safety information showing that musculoskeletal events are rare and reversible [10–13]. Currently, the United States Food and Drug Administration (US FDA)-approved pediatric indications for FQs include inhalational anthrax, complicated urinary tract infections, and pyelonephritis for ciprofloxacin (CF), and inhalational anthrax for levofloxacin (LF) [27, 28]. Some European countries also allow the use of CF in children under certain indications [29].

To our knowledge, no quantitative studies have been conducted to quantify national prescription volume of fluoroquinolone in children. Although one study has assessed FQ use in Korean pediatric patients, it used patient sample data and short study period of two years, and thus limited in evaluating the overall, sustained impact of the nationwide intervention [30]. Thus, the aims of our study were 1) to quantify the national pediatric use of fluoroquinolones and 2) to evaluate the sustained as well as immediate effect of nationwide implementation of age restriction on fluoroquinolone use by analyzing the changing rates and patterns of prescription over 9 years. Based on the understanding of the national reimbursement policy, where the prescription of a contraindicated drug included in the list may be restrictively reimbursed, we hypothesized that the classification of FQ as drug contraindicated for pediatric use would result in a dramatic reduction in the number of FQ prescriptions.

## Materials and methods

### Data source

The Health Insurance Review and Assessment Service (HIRA), a Korean regulatory agency, is responsible for the review and assessment of reimbursement and plays a leading role in managing the DUR. The entire Korean population, with the exception of the 3% covered under medical aid, is enrolled in the national health insurance system, which achieved universal coverage of all citizens in 1989 [31]. The HIRA database contains nationwide information from medical facilities and pharmacies on patient demographics, diagnoses, prescriptions, and healthcare service providers. For reimbursement, the patient’s diagnosis must be confirmed by physicians, and the Korean National Health Insurance agency reviews it against the eligibility criteria, before submitting claims to the HIRA claim database. Thus, these data are considered reliable [32]. The ethics approval was obtained from the Institutional Review Boards of Chungnam National University and Chung-Ang University.

### Study period and population

Data were collected from 2007 to 2015. Due to the implementation of the DUR age restriction on FQ use in December 2009, the time periods were separated as follows for purposes of comparison: “pre-DUR:” 2007 to 2009; “post-DUR:” 2010 to 2015. Both male and female patients who met the following criteria at the time of prescription were included: (i) younger than 18 years, (ii) visited a medical facility from 2007 to 2015, and (iii) received a prescription for systemic CF or LF. Data on topical FQ use were excluded.

### Data collection and assessment

Drug information, such as the generic name of the drug, prescription date, duration, and route of administration were collected. Gender and age were determined at the time of hospital admission or on the first outpatient prescription. To evaluate the rationale behind prescription as well as its patterns, we analyzed the International Classification of Diseases, tenth revision (ICD-10) diagnostic codes and the specialties of prescribers. To determine which medical facility the DUR implementation had a greater impact on, the number of FQ prescriptions prescribed in each medical facility was analyzed. The medical facility at which FQ was prescribed was classified according to the domestic hospital classification system: clinic, hospital, general hospital, or tertiary referral hospital. As all personal data were de-identified, patient information and healthcare service information could not be matched.

To assess other factors that may have impacted pediatric FQ prescriptions, such as drug shortages and overall changes in pediatric antibiotic prescription patterns, data on the prescription volumes of five commonly prescribed pediatric antibiotics (amoxicillin, amoxicillin and clavulanate in combination, cefaclor, cefixime, and clarithromycin; selection based on literature review [33–35]) as well as FQs prescribed in adults were collected using the HIRA National Patients Sample (HIRA-NPS) database [36].

### Statistical analysis

The annual prescription rate of FQ per 100,000 person-years was calculated as the number of patients prescribed FQ divided by the number of all Korean residents in the same age group, which was obtained from the national statistics data of 2010 [37]. Categorical variables were expressed as frequencies and proportions. A chi-square (χ^2^) test was performed to assess changes in prescription patterns after DUR implementation with regard to diagnostic codes, types of medical facility, and age groups. An autoregressive model was used to predict the prescription pattern if intervention had not occurred. The model coefficient and covariance were estimated based on the maximum-likelihood method. *P* < 0.05 were considered statistically significant, and all statistical analyses were performed using SAS version 9.3 (SAS Institute Inc., Cary, NC).

## Results

### Characteristics of the study population and number of FQ prescriptions

Between 2007 and 2015, a total of 505,859 pediatric patients younger than 18 years received systemic FQs: 297,054 received CF, and 208,805 received LF (Table 1). No significant gender differences were observed between the pre-DUR and post-DUR groups (CF: *P* = 0.253; LF: *P* = 0.126); however, a significant increase in the mean age of patients was observed after the implementation of DUR (mean age ± SD; CF: 13.31 ± 4.14 vs. 14.36 ± 3.63 years, *P* < 0.001; LF: 14.15 ± 3.10 vs. 14.91± 3.31 years, *P* < 0.001).

**Table 1:** Demographic characteristics of pediatric patients who received systemic fluoroquinolone treatment.

Characteristics (No, %)	Ciprofloxacin				Levofloxacin			
	Inpatient		Outpatient		Inpatient		Outpatient	
	Pre-DUR	Post-DUR	Pre-DUR	Post-DUR	Pre-DUR	Post-DUR	Pre-DUR	Post-DUR
	(n = 19284)	(n = 7553)	(n = 263513)	(n = 6704)	(n = 7435)	(n = 2474)	(n = 193955)	(n = 4941)
**Age**								
** <4 wks**	31 (0.2)	21 (0.3)	76 (0.0)	0 (0)	0 (0)	10 (0.4)	35 (0)	1 (0)
** 4 wks–11 mos**	143 (0.7)	168 (2.2)	3890 (1.5)	18 (0.3)	11 (0.1)	35 (1.4)	348 (0.2)	7 (0.1)
** 1–2 yrs**	482 (2.5)	399 (5.3)	7794 (3.0)	36 (0.5)	39 (0.5)	35 (1.4)	1809 (0.9)	35 (0.7)
** 3–5 yrs**	980 (5.1)	616 (8.2)	11816 (4.5)	90 (2.4)	60 (0.8)	51 (2.1)	5121 (2.6)	99 (2.0)
** 6–11 yrs**	2233 (11.6)	771 (10.2)	54743 (20.8)	484 (7.2)	711 (9.6)	174 (7.0)	35015 (18.1)	312 (6.3)
** 12–17 yrs**	15415 (79.9)	5578 (73.9)	185194 (70.3)	6076 (90.6)	6614 (89.0)	2169 (87.7)	151627 (78.2)	4487 (90.8)
**Gender**								
** Male**	10663 (55.3)	4278 (56.6)	137684 (52.2)	3131 (46.7)	4531 (60.9)	1400 (56.6)	100554 (51.8)	2398 (48.5)

Abbreviations: DUR, drug utilization review; wks, weeks; mos, months; yrs, years.

### Annual FQ prescription rate

**Fig 1:**
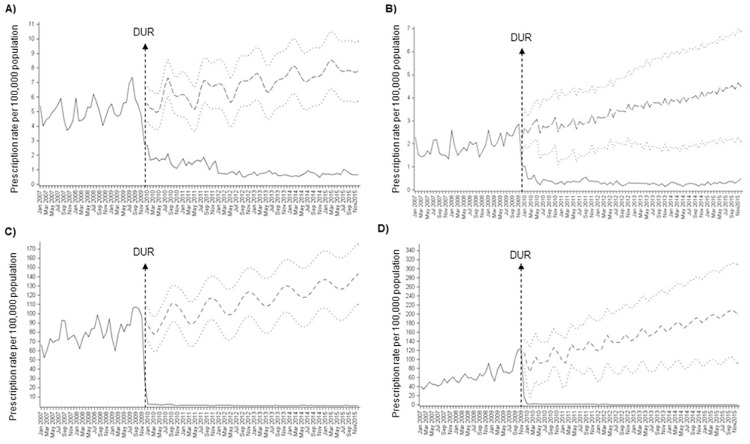
Impact of drug utilization review (DUR) intervention on the monthly fluoroquinolone prescription rates per 100,000 children younger than 18 years old. (A) monthly prescription rate of CF in inpatient setting; (B) monthly prescription rate of LF in inpatient setting; (C) monthly prescription rate of CF in outpatient setting; (D) monthly prescription rate of LF in outpatient setting. Solid lines represent observed values, dashed lines represent predicted values and dotted lines represent 95% confidence intervals of predicted values. Abbreviations: CF, ciprofloxacin; LF, levofloxacin; DUR, drug utilization review.

**Fig 2:**
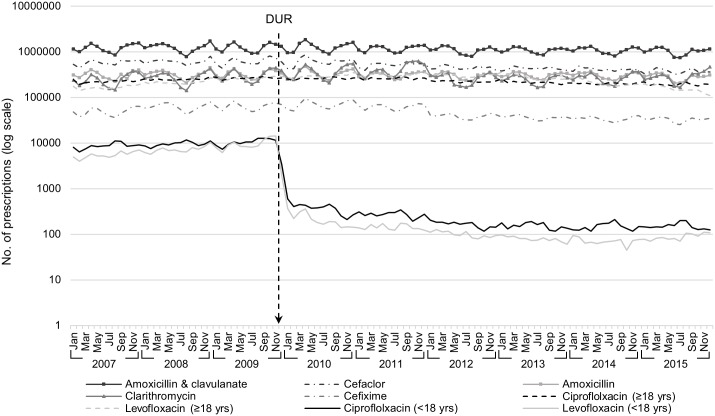
Prescription of fluoroquinolones and other antibiotics to children and prescription of fluoroquinolone to adults (log scale).

The monthly FQ prescription rate decreased significantly after the DUR implementation (Fig 1). The mean annual pre-DUR prescription rate of CF decreased from 840 to 21 per 100,000 person-years (relative reduction: 97.5%) and LF from 598 to 11 per 100,000 person-years (relative reduction: 98.2%). A greater reduction in the FQ prescription rate was observed in the outpatient (CF: from 782 to 10 per 100,000 person-years, 98.7%; LF: from 576 to 7 per 100,000 person-years, 98.8%) than in the inpatient group (CF: from 57 to 11 per 100,000 person-years, 80.7%; LF: from 22 to 4 per 100,000 person-years, 81.8%). Prescription rate of other commonly prescribed antibiotics in children and prescription of FQs in adults remained stable during the study period (Fig 2).

### Claimed diagnostic codes and specialties of prescribers

The top five most common diagnostic codes under which FQ was prescribed in children are shown in S1 Table. The most commonly used diagnostic code in the inpatient group remained unchanged after DUR (CF: certain infectious and parasitic diseases [A00–B99]; LF: diseases of the respiratory system [J00–J99]), and only minor changes occurred in the other top five diagnostic codes. In the outpatient setting, respiratory system diseases (J00-J99) were the most prevalent diagnostic codes associated with prescriptions of both CF and LF in the pre-DUR group. However, after DUR, significant changes in the diagnostic codes were observed with CF most commonly prescribed for certain infectious and parasitic diseases (A00–B99), and LF for diseases of the genitourinary system (N00–N99).

Furthermore, we analyzed the specialties of the physicians who prescribed FQ. The top five specialties of physicians who prescribed FQ in children are shown in Table 2. The most common specialties that prescribed FQs in the inpatient setting remained the same (both CF and LF: ‘Internal medicine’), and only minor changes between the pre-DUR and post-DUR periods were observed in the FQ prescription rates by the other top-ranked specialties. Notably, ‘Pediatrics’ ranked second in post-DUR LF inpatient prescriptions despite not being included in top five in pre-DUR. In the outpatient setting, significant changes in the distribution of FQ prescriptions across specialties were observed after DUR. For CF, ‘Obstetrics & Gynecology’ and ‘Surgery’ became newly ranked in top five after DUR replacing ‘Otolaryngology’ and ‘Ophthalmology’. Similarly, for LF, ‘Urology’ and ‘Obstetrics & Gynecology’ became newly ranked in top five after DUR replacing ‘Ophthalmology’ and ‘Pediatrics’.

**Table 2:** Five most common medical specialties of physicians prescribing pediatric fluoroquinolone prescriptions.

	Prescriber specialty, N (%)			
**CF inpatient**	Rank	Pre-DUR (n = 19284)	Rank	Post-DUR (n = 7553)
	1	Internal Medicine: 9446 (49.0)	1	Internal Medicine: 2687 (35.6)
	2	Surgery: 2080 (10.8)	2	Otolaryngology: 1252 (16.6)
	3	Otolaryngology: 2047 (10.6)	3	Pediatrics: 1180 (15.6)
	4	Orthopedics: 1958 (10.2)	4	Orthopedics: 885 (11.7)
	5	Pediatrics: 1191 (6.2)	5	Surgery: 624 (8.3)
**CF outpatient**	Rank	Pre-DUR (n = 263513)	Rank	Post-DUR (n = 6704)
	1	Internal Medicine: 90469 (34.3)	1	Internal Medicine: 3005 (44.8)
	2	Otolaryngology: 64643 (24.5)	2	Urology: 678 (10.1)
	3	Pediatrics: 30836 (11.7)	3	Obstetrics & Gynecology: 627 (9.4)
	4	Ophthalmology: 24350 (9.2)	4	Pediatrics: 524 (7.8)
	5	Urology: 12358 (4.7)	5	Surgery: 322 (4.8)
**LF inpatient**	Rank	Pre-DUR (n = 7435)	Rank	Post-DUR (n = 2474)
	1	Internal Medicine: 2629 (35.4)	1	Internal Medicine: 1024 (41.4)
	2	Surgery: 1039 (14.0)	2	Pediatrics: 402 (16.2)
	3	Orthopedics: 913 (12.3)	3	Orthopedics: 252 (10.2)
	4	Otolaryngology: 849 (11.4)	4	Otolaryngology: 223 (9.0)
	5	Urology: 446 (6.0)	5	Surgery: 207 (8.4)
**LF outpatient**	Rank	Pre-DUR (n = 193955)	Rank	Post-DUR (n = 4941)
	1	Internal Medicine: 57390 (29.6)	1	Internal Medicine: 1575 (31.9)
	2	Otolaryngology: 30020 (15.5)	2	Urology: 822 (16.6)
	3	Ophthalmology: 28743 (14.8)	3	Obstetrics & Gynecology: 564 (11.4)
	4	Pediatrics: 22870 (11.8)	4	Otolaryngology: 356 (7.2)
	5	Dermatology: 16611 (8.6)	5	Dermatology: 324 (6.6)

Abbreviations: CF, ciprofloxacin; LF, levofloxacin; DUR, drug utilization review

### Medical facility distribution of FQ prescriptions

**Fig 3:**
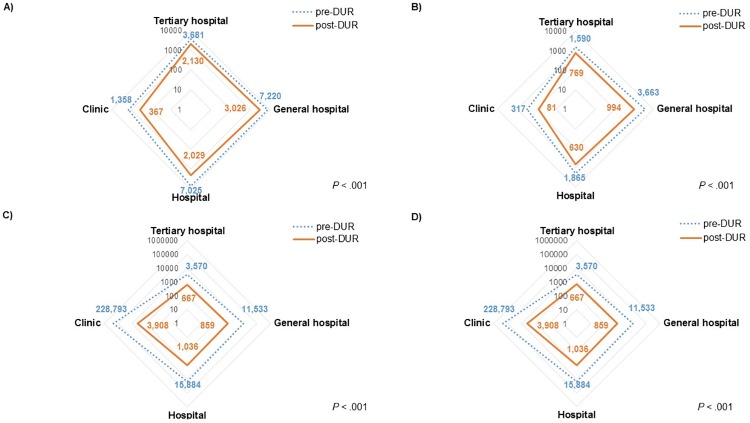
Distribution of pediatric fluoroquinolone prescriptions in medical facilities before and after implementation of drug utilization review. (A) distribution of CF prescriptions in medical facilities in inpatient setting, pre- and post-DUR (log scale); (B) distribution of LF prescriptions in medical facilities in inpatient setting, pre- and post-DUR (log scale); (C) distribution of CF prescriptions in medical facilities in outpatient setting, pre- and post-DUR (log scale); (D) distribution of LF prescriptions in medical facilities in outpatient setting, pre- and post-DUR (log scale).

**Fig 4:**
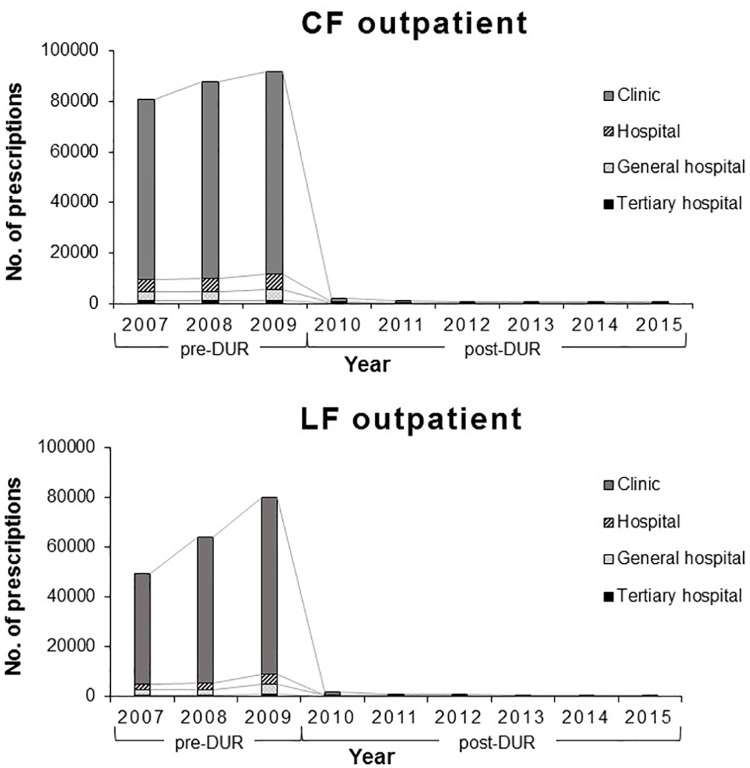
Yearly distribution of pediatric fluoroquinolone prescriptions in medical facilities in outpatient setting. Abbreviations: CF, ciprofloxacin; LF, levofloxacin; DUR, drug utilization review.

Significant changes in the distribution of FQ prescriptions by type of medical facility were observed after DUR implementation, (Fig 3; CF: inpatient *P* < .001, outpatient *P* < .001; LF: inpatient *P* < .001, outpatient *P* < .001). Such changes were more dramatic in the outpatient setting than in the inpatient setting and the changes in the yearly distribution of CF and LF prescriptions in the outpatient settings are shown in Fig 4. The proportion of FQ prescriptions issued by private clinics declined significantly after DUR, whereas those issued by other medical facilities, hospital, general hospital an tertiary hospital, increased.

## Discussion

Our study is first to assess the national off-label prescription of FQ in children, as no approved indications exist for FQ in South Korea. Prescription volume of FQ in all healthcare settings was observed over 9 years using a large-scale national database. Fluoroquinolone use declined substantially and immediately after the implementation of age restriction under DUR. The effect has been sustained for six years since implementation.

The nationwide incidence of FQ off-label prescription in children declined from 0.840% to 0.021% for CF and from 0.598% to 0.011% for LF after the regulatory action. A study conducted in British Columbia, Canada showed an incidence ranging between 0.06% and 0.09%, which is closer to the incidence after DUR implementation in South Korea [9]. Due to the lack of nationwide studies in other countries, direct comparisons are not possible. However, the pediatric FQ use in South Korea can be thought to be high as there are no approved indications in South Korea. The incidence of FQ prescription in the ambulatory setting was found to be higher than in the inpatient setting before the regulatory action, and a greater reduction occurred in the ambulatory setting than in the inpatient setting.

The pattern of the diagnostic codes associated with FQ prescription changed markedly after DUR implementation, especially in the outpatient setting. Before the implementation of DUR, ‘Diseases of the respiratory system (J00–J99)’ were the most commonly used diagnostic codes for both CF and LF outpatient prescriptions. However, after DUR implementation, ‘Certain infectious and parasitic diseases (A00–B99)’ and ‘Diseases of the genitourinary system (N00–N99)’ were the most commonly used diagnostic codes for CF and LF, respectively. In regards to medical specialties, ‘Internal medicine’ remained to be the most common medical specialty of physicians who prescribed FQs to children in both inpatient and outpatient settings for both CF and LF after DUR implementation. Prescribers with internal medicine as specialty are considered primary care physicians in Korea and internal medicine is a common specialty visited by patients of all ages [38]. The distribution of medical facilities at which FQs were prescribed also shifted significantly. After DUR implementation, the proportion of FQ prescriptions issued in clinics and primary care settings declined significantly; however, the proportion of prescriptions issued by referral hospitals increased, and such change was more dramatic in the ambulatory outpatient settings. The high volume of FQ prescriptions in primary care setting before DUR implementation is especially alarming, as unnecessary prescription of antibiotics frequently occur in an ambulatory setting [39, 40]. Additionally, current recommendations for pediatric FQ use are restricted to situations were no safe and effective alternative is available and the infection is caused by multidrug-resistant pathogens likely requiring inpatient treatment. Therefore, the shift in FQ prescription from primary care settings to referral hospitals, especially in the outpatient setting, is promising. The volume of prescriptions of other antibiotics for pediatric patients, as well as that of FQ to adults, was stable during the study period from 2009 to 2015, confirming that no other factors such as drug shortages or overall changes in pediatric antibiotic prescription are attributable to such significant reduction in FQ prescription volume in children.

Educational strategies, community-wide campaigns, and outpatient antimicrobial stewardship interventions often fail to exert a statistically significant and sustained effect on the issuance of antibiotic prescriptions [41–45]. Antimicrobial stewardship interventions by hospitals are commonly implemented to control for inappropriate use of antibiotics. However, in ambulatory settings, especially those involving primary care, these interventions or policies are rarely applied, which may be a reason behind the excessive antibiotic use in these settings. FQs have been suggested as the drugs of choice in treating complicated infections caused by drug-resistant pathogens in children [14, 15]. However, on the other hand, the inappropriate prescription of antibiotics is the main factor driving the emergence and spread of antibiotic resistance, and increasing FQ resistance is also of concern in pediatric populations [16, 46–50]. Therefore, it is important to promote judicious prescribing of FQ in children, only in situations where its use is required. The DUR system implemented in South Korea can contribute to such movement by encouraging reassessment of selecting FQ for use in pediatric patients.

The outcomes of the intervention under DUR program are not only positive in terms of reducing resistance but also limiting potential toxicity in pediatrics. Fluoroquinolones were initially restricted for use in children due to potential for cartilage toxicity. Recently, US FDA issued a FDA Drug Safety Communication recommending restriction of fluoroquinolones in adults due to side effects involving the tendons, muscles and joints [51]. Thus, it may be necessary to limit fluoroquinolones to absolutely needed conditions in both adults and children. Regulatory interventions, like DUR, may be required in other countries to effectively and sustainably restrict use of fluoroquinolone antibiotics. Additionally, it may be valuable to assess the impact of other public health interventions, such as the recent issue of the US FDA Drug Safety Communication [51], although, to the best of our knowledge, the interventions implemented in other systems are likely to be much less invasive the intervention under Korean DUR system.

Our results should be interpreted with caution. Claims data on individual-level do not contain information such as exact diagnosis, isolated pathogens, or antimicrobial susceptibility. Although diagnostic codes were collected for all prescribing events, the question of whether the changes in pediatric FQ prescription patterns align with the recommendations in the literature or with its approval status in other countries cannot be answered. Thus, we were unable to assess the appropriateness of any particular FQ prescription; therefore, it was not possible to determine whether most of the eliminated prescriptions were inappropriate. Also, domestic and international antimicrobial stewardship initiatives conducted during our study period may have influenced the results of the study and confounding factor from unmeasurable variables could not be excluded. Although we attempted to exclude the possible effect of drug shortages and overall changes in pediatric antibiotic prescription patterns by analyzing the prescription volume of five commonly prescribed antibiotics, it was not possible to determine which antibiotics had been used in place of FQ. Despite such limitations, it is evident that FQ use in children decreased in South Korea after DUR implementation and further studies on how the regulatory action influenced antibiotic prescribing and clinical outcomes are required.

This study is first to analyze the nationwide pediatric prescription of FQ. Age restriction under the DUR system resulted in an immediate, dramatic decline in FQ prescription and which effect was sustained afterwards. Regulatory action via the national DUR system appears to be especially effective in the ambulatory setting, promoting more judicious use of FQ as part of antimicrobial stewardship on a national level. Therefore, the potential use of similar regulatory intervention to control inappropriate and excessive use of certain medications in other countries, states or institutions appear promising.

## Supporting information

S1 Table Five most commonly used diagnostic codes of pediatric fluoroquinolone prescriptions. (PDF)
