# Supplementary material for: Pediatric fluoroquinolone prescription in South Korea before and after a regulatory intervention: A nationwide study, 2007-2015
Source: PLoS One. 2017 May 17;12(5):e0176420. doi: 10.1371/journal.pone.0176420 (PMC5435163; doi:10.1371/journal.pone.0176420)
Supplement: S1 Table — (PDF) [file pone.0176420.s001.pdf]

S1 Table. Five most commonly used diagnostic codes of pediatric fluoroquinolone prescriptions.

	Diagnostic codes, No. (%)			
	Rank	Pre-DUR (n = 19284)	Rank	Post-DUR (n = 7553)
**CF inpatient**	1	Certain infectious and parasitic diseases (A00–B99): 5845 (30.3)	1	Certain infectious and parasitic diseases (A00–B99): 1940 (25.7)
	2	Diseases of the digestive system (K00–K93): 2798 (14.5)	2	Diseases of the ear and mastoid process (H60-H95): 977 (12.9)
	3	Diseases of the respiratory system (J00–J99): 2229 (11.6)	3	Injury, poisoning and certain other consequences of external causes (S00–T98): 852 (11.3)
	4	Injury, poisoning and certain other consequences of external causes (S00–T98): 1861 (9.7)	4	Diseases of the digestive system (K00–K93): 794 (10.5)
	5	Diseases of the eye and adnexa (H00–H59): 1579 (8.2)	5	Diseases of the respiratory system (J00–J99): 661 (8.8)
	Rank	Pre-DUR (n = 263513)	Rank	Post-DUR (n = 6704)
**CF outpatient**	1	Diseases of the respiratory system (J00–J99): 75683 (28.7)	1	Certain infectious and parasitic diseases (A00–B99): 2257 (33.7)
	2	Diseases of the ear and mastoid process (H60-H95): 53250 (20.2)	2	Diseases of the genitourinary system (N00–N99): 1544 (23.0)
	3	Certain infectious and parasitic diseases (A00–B99): 50145 (19.0)	3	Diseases of the digestive system (K00–K93): 972 (14.5)
	4	Diseases of the eye and adnexa (H00–H59): 19052 (7.2)	4	Diseases of the respiratory system (J00–J99): 721 (10.8)
	5	Diseases of the genitourinary system (N00–N99): 18487 (7.0)	5	Symptoms, signs and abnormal clinical and laboratory findings, not elsewhere classified (R00–R99): 234 (3.5)

	Rank	Pre-DUR (n = 7435)	Rank	Post-DUR (n = 2474)
**LF inpatient**	1	Diseases of the respiratory system (J00–J99): 1898 (25.5)	1	Diseases of the respiratory system (J00–J99): 758 (30.6)
	2	Injury, poisoning and certain other consequences of external causes (S00–T98): 1113 (15.0)	2	Certain infectious and parasitic diseases (A00–B99): 361 (14.6)
	3	Certain infectious and parasitic diseases (A00–B99): 1090 (14.7)	3	Injury, poisoning and certain other consequences of external causes (S00–T98): 254 (10.3)
	4	Diseases of the digestive system (K00–K93): 1069 (14.4)	4	Neoplasms (C00–D48): 191 (7.7)
	5	Diseases of the genitourinary system (N00–N99): 575 (7.7)	5	Diseases of the genitourinary system (N00–N99): 163 (6.6)
	Rank	Pre-DUR (n = 193955)	Rank	Post-DUR (n = 4941)
**LF outpatient**	1	Diseases of the respiratory system (J00–J99): 85329 (44.0)	1	Diseases of the genitourinary system (N00–N99): 1378 (27.9)
	2	Diseases of the ear and mastoid process (H60-H95): 32196 (16.6)	2	Diseases of the respiratory system (J00–J99): 1206 (24.4)
	3	Diseases of the skin and subcutaneous tissue (L00–L99): 19492 (10.0)	3	Certain infectious and parasitic diseases (A00–B99): 670 (13.6)
	4	Certain infectious and parasitic diseases (A00–B99): 16758 (8.6)	4	Diseases of the ear and mastoid process (H60-H95): 446 (9.0)
	5	Diseases of the genitourinary system (N00–N99): 16255 (8.4)	5	Diseases of the skin and subcutaneous tissue (L00–L99): 403 (8.2)

Abbreviations: CF, ciprofloxacin; LF, levofloxacin; DUR, drug utilization review
